# Analysis and Hazard Assessment of Potentially Toxic Metals in Petroleum Hydrocarbon-Contaminated Soils Around Transformer Installation Areas

**DOI:** 10.5696/2156-9614-9.24.191213

**Published:** 2019-12-06

**Authors:** Godswill E. Akhigbe, Festus M. Adebiyi, Nkem Torimiro

**Affiliations:** 1 Department of Chemistry, Obafemi Awolowo University, lle-Ife, Nigeria; 2 Department of Chemical Sciences, McPherson University, Seriki-Sotayo, Nigeria; 3 Department of Microbiology, Obafemi Awolowo University, lle-Ife, Nigeria

**Keywords:** contamination, AAS, hazard assessment, toxic metal, petroleum hydrocarbon, soil

## Abstract

**Background.:**

Soil contamination resulting from the use and handling of petrochemicals and other petroleum products during power generation activities is an increasing global concern due to its adverse impact on the ecosystem.

**Objectives.:**

The present study was carried out to determine the concentrations and speciation of potentially toxic metals in oil-contaminated soils around transformer installation areas in Ile-Ife, Nigeria, and to confirm soil pollution levels with hazard quotient and hazard index analysis.

**Methods.:**

Soils from the transformer oil-contaminated and uncontaminated (control) areas were collected at 0–15 cm and 15–30 cm depths and analyzed for heavy metal concentrations using atomic absorption spectrometry. The metals were fractionated and their hazard evaluated to confirm the pollution level of the contaminated soils.

**Results.:**

The concentrations of cadmium (Cd), chromium (Cr), copper (Cu), iron (Fe), manganese (Mn), nickel (Ni), lead (Pb) and zinc (Zn) in the two sets of oil-impacted soils were higher than in the control soils. The metals also had relatively moderate bioavailability and mobility potential with more of the proportion retained in the residual fraction. Chronic daily intake (CDI) of the metals increased in the order of: Cd < Cr < Pb < Ni < Mn < Cu < Zn < Fe, while chronic daily intake risk exposure pathway followed the order of: CDI_inhalation_ < CDI_dermal_ < CDI_ingestion._

**Conclusions.:**

The study concluded that the concentrations of the metals were within permissible limits, but the chronic daily dosage was significant and may pose a health hazard to humans with long term exposure to these heavy metal contaminants.

**Competing Interests.:**

The authors declare no competing financial interests.

## Introduction

The increasing global demand for petroleum and petroleum products such as transformer oil for energy generation has led to an increase in ecosystem contamination. 1,2 This has adverse effects on soil microflora, fauna, soil fertility, microorganisms, and humans, leads to soil nitrogen deficiency, and an imbalance in essential minerals in soil.^3^,^4^ Environmental contaminants arising from spillage of petroleum hydrocarbon products and their derivatives usually involve heavy metals.^3^,^5^ If spills are not properly managed, they can lead to the release of recalcitrant and harmful environmental pollutants such as heavy metals into underground water by leaching into subsurface soil layers and surface runoff.

Soil has the capacity to retain heavy metals in both solid and solution phases, these metals are immobilized in the solid phase as they adsorb soil components, and they are mobile in the solution phase.^6^,^7^ The metal ions in the solid phase may become available and mobile if there is a change in soil pH. Soil pH can influence oxidation-reduction potential and cation exchange capacity. Heavy metals are more mobile and bioavailable in the solution phase than in the solid phase.^7^ Determining the total metal concentration in soils helps to estimate the heavy metal burden, enrichment, depletion and pollution, which is useful in geochemical studies. However, it does not provide information on the chemical forms in which the metals exist or on the bioavailability and mobility of the metals, which is helpful in evaluating the risk associated with the presence of metals in the environment in agricultural studies.^8–10^ The major routes through which heavy metals in soils can reach humans and cause health hazards are through direct ingestion, skin contact, and inhalation.^11^

The objective of the present study was to determine the concentrations and speciation of potentially toxic metals in the oil-contaminated soils around transformer installation areas in Ile-Ife, Nigeria, as well as to evaluate the exposure hazard of the contaminated soils for nearby residents in the study area by evaluating the hazard quotient and index.

## Methods

**Figure 1:**
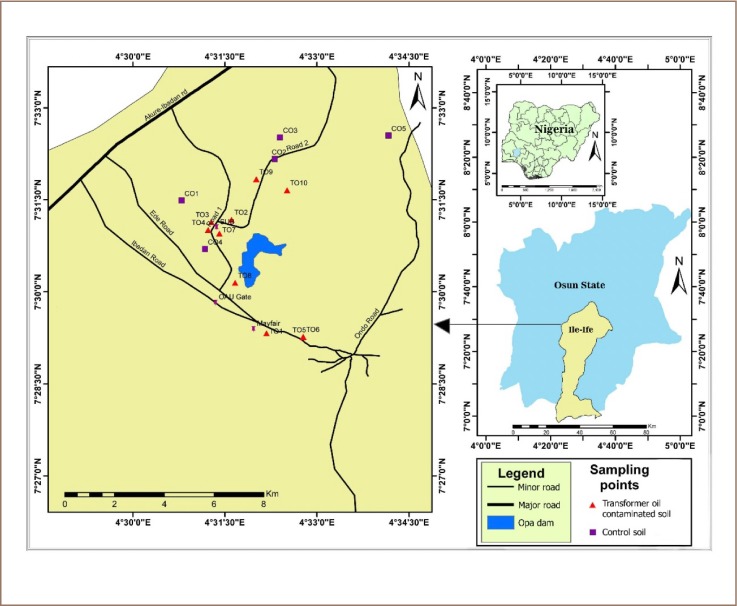
Geographical map of the study area showing the various sampling points

A map of the study area showing the various sampling points is presented in Figure 1, while Table 1 contains the coordinates of the sampling locations. The study area, Ile-Ife, is located between latitudes 7°26′ N and 7°34′ N and longitudes 4°28′ E and 4°34′ E. It is an ancient town located in Osun State, within the southwestern geopolitical zone of Nigeria. It is a commercialized, university town with few industries that is surrounded by rural settlements from which it gets most of its food supply. The predominant economic mainstay of the inhabitants includes small-scale agricultural practices, timber processing and commercial transportation activities.^12^

**Table 1:** Sampling Location Coordinates

**Sampling location code**	**Longitude**	**Latitude**
TO1	7′29′19.43235	4′32′10.49592
TO2	7′31′10.68996	4′31′36.40725
TO3	7′31′8.37194	4′31′16.74539
TO4	7′31 ′0.65289	4′31′ 13.57546
T05	7′29′15.82701	4′32′46.47929
TO6	7′29′16.21584	4′32′46.49487
TO7	7′30′57.04646	4′31′24.51671
TO8	7′30′8.87675	4′31 ′40.01416
TO9	7′31′50.29585	4′32′0.64311
TO10	7′31′39.462	4′32′30.74951
CO1	7′31′29.42412	4′30′47.61068
CO2	7′32′9.94604	4′32′ 18.3384
CO3	7′32′31.14634	4′32′23.83918
CO4	7′30′41.896681	4′31′10.47023
CO5	7′32′33.37958	4′31′9.69999

Abbreviations: TO, transformer oil-contaminated soil sampling locations; CO, control soil sampling location

### Sample collection

Ten samples of soils contaminated with transformer oil were collected randomly from the various transformer installation areas in Ile-Ife, Osun State, Nigeria. Five control soil samples were collected from soils with little or no anthropogenic influences about 100 m from the contaminated site. All samples were collected at two different sampling depths of 0–15 cm (surface soil) and 15–30 cm (sub-surface soil) using a graduated Dutch hand-held auger. The soil samples were prepared for metal analysis using the method described by Asubiojo *et al*.^13^

**Abbreviations**
*AAS*: Atomic absorption spectrometry
*CDI*: Chronic daily intake
*HI*: Hazard index
*HNO_3_*: Nitric acid
*HQ*: Hazard quotient
*I_Geo_*: Geo-accumulation index
*PI*: Pollution index
*THI*: Total hazard index
*THQ*: Total hazard quotient
*USEPA*: United States Environmental Protection Agency

### Quality assurance and control protocols

In order to determine the precision of the analytical procedure and results, quality assurance and control were carried out. Sample bottles and apparatus used for the analysis were pre-treated using the method described by Oyewole and Adebiyi.^14^

### Recovery analysis

Recovery analysis was conducted to determine the precision and control of the analytical procedures used for the atomic absorption spectrometry (AAS) analysis in this study. One (1) g of the sample in a Teflon beaker was spiked with a known concentration of lead (II) ion (Pb^2+^), manganese (II) ion (Mn^2+^), copper (II) ion (Cu^2+^), and zinc (II) ion (Zn^2+^) solutions. Recovery analysis carried out with methods described by Ogunfowokan *et al*.^15^

### Total digestion

A 0.5 g portion of the pulverized dried soil samples was digested in a Teflon beaker using 6 ml concentrated nitric acid (HNO_3_) and 2 ml concentrated hydrochloric acid in the ratio 3:1 (aqua regia) as reported by Oyewole and Adebiyi.^14^

### Speciation of heavy metals

**Figure 2:**
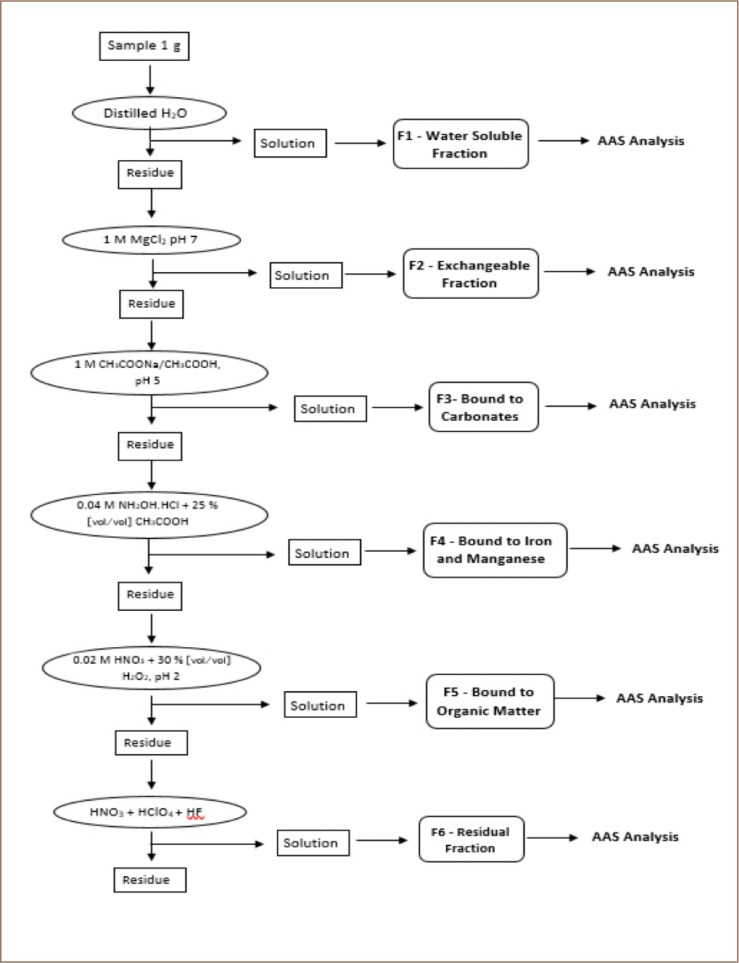
Sequential extraction of metals from the soil samples

The method used for the extract and partitions of metals (lead (Pb), cadmium (Cd), nickel (Ni), iron (Fe), chromium (Cr), copper (Cu), zinc (Zn), and manganese (Mn)), into water-soluble, exchangeable, carbonate-bound, Fe - Mn oxides bound, organic bound and residual fractions was proposed by Tessier *et al.* and adapted by Oyewole and Adebiyi.^14^,^16^ A flow chart describing the sequential extraction of metals from the soil samples is shown in Figure 2.

A summary of the metal speciation procedure is described below.

### F1—Water-soluble fraction

A total of 0.5 g of air-dried soil samples was mixed with 10 ml of distilled water and continuously agitated for 1 hour using a mechanical shaker. It was then centrifuged for 15 minutes. The supernatant was decanted and made up to 25 ml with distilled water and stored in a clean plastic bottle.

### F2—Exchangeable fraction

The residue from F1 extraction was extracted at room temperature for 1 hour with 10 ml of 1 M magnesium chloride solution at pH 7 with continuous agitation for 1 hour. The resultant extract was decanted and made up to 25 ml with distilled water, and thereafter, stored in a clean plastic bottle.

### F3—Acid extractable carbonates bound fraction

The residue of F2 was extracted with 10 ml of 1 M sodium acetate/acetic acid buffer at pH 5 for 5 hours with continuous agitation at room temperature. The extracted metal solution was decanted from the residual sand and made to 25 ml using distilled water, and then stored in a clean plastic bottle.

### F4—Reducible Fe-Mn oxides and hydroxide fraction

The residue from F3 was extracted under mild reducing conditions with 10 ml of 0.04 M hydroxylamine hydrochloride in 25% (vol/vol) acetic acid with agitation at 96°C in a water bath for 6 hours with occasional agitation. The extracted metal solution was decanted from the residual sediment and made up to 25 ml using distilled water, and then stored in a clean plastic bottle.

### F5—Oxidizable organic matter and sulfide bound fraction

The residue from F4 was oxidized using a solution containing 7.5 ml of 0.02 M HNO_3_ and 12.5 ml of hydrogen peroxide (30%, vol/vol), at pH 2. The mixture of the solution and residue from F4 was heated to 85°C in a water bath for 2 hours with occasional agitation and allowed to cool. Another 7.5 ml of hydrogen peroxide (30%), adjusted to pH 2 with HNO_3_, was then added. The mixture was heated again at 85°C for 3 hours with occasional agitation and allowed to cool down. After cooling, 12.5 ml of 3.2 M ammonium acetate in HNO_3_ (20%, vol/vol) was added to prevent the adsorption of extracted metals into the oxidized sediments, and the mixture was diluted to a final volume of 25 ml with de-ionized water followed by agitation for 30 minutes. The extracted metal solution was decanted from the residual sediment which was used for the next extraction.

### F6—Residual or inert fraction

Residue from F5 was oven-dried at 105°C. Digestion was carried out with a mixture of 7.5 ml concentration of HNO_3_ (70% wt/wt), 5 ml concentration of hydrofluoric acid (40% wt/wt) and 5 ml concentration of perchloric acid (60% wt/wt) in Teflon beakers. The fractions were taken for AAS analysis.

### Mobility factor determination

Mobility factor, an index of potential mobility of metal ions in soil was determined on the basis of the absolute and relative values of fractions (F1, F2, and F3) weakly bound to soil components using the relationship as described in previous studies *(Equation 1)*.^7^,^17^

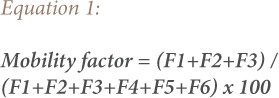
where, F1 = water-soluble fraction, F2 = exchangeable fraction, F3 = carbonate bound fraction, F4 = Fe-Mn bound fraction, F5 = organic bound fraction, and F6 = residual or inert fraction.

### Data analysis

For the interpretation of the heavy metals concentrations, cluster analysis, analysis of variance, t-test analysis, geo-accumulation and pollution index statistical methods described by Oyewole and Adebiyi were used.^14^

### Potential human health hazard assessment

In order to calculate the soil heavy metal exposure risk to children and adults in the present study, the United States Environmental Protection Agency (USEPA) health risk assessment model was adopted.^18^ It was used for the estimation of the bio-accessibility of heavy metals and their impact on humans and the environment. The parameters used in estimating human exposure risk to heavy metals in Equations 2, 3, and 4 are based on criteria of the USEPA and the Department of Environmental Affairs, South Africa.^18^,^19^

Using Equations 2, 3, and 4, the chronic daily intake (CDI) mg/kg/day was used to evaluate exposure to heavy metals in the soil.^18^,^20^,^21^

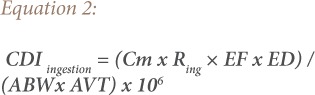


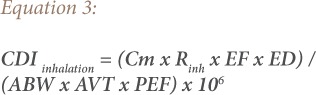


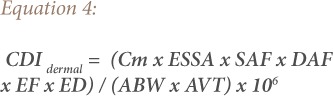
where,

CDI (mg kg^−1^ day^−1^) is the chronic daily dose of heavy metals in the soil through ingestion (CDI*_ing_*), inhalation (CDI*_inh_*) and dermal contact (CDI*_dermal_*); Cm (mean concentration of each analyzed heavy metals) is the exposure point concentration of heavy metals in the soil (mg kg^−1^); R*ing* (soil ingestion rate) at 200 mg day^−1^ for children (1–6 years) and 100 mg day^−1^ for adults; R*inh* (inhalation rate) at 10 m^3^ day^−1^ for children and 20 m^3^ day^−1^ for adults; EF (exposure frequency) = 350 day years^−1^ for children and adults; ED (exposure duration) = 6 years for children and 30 years for adults; ABW (average body weight) = 15 kg for children and 70 kg for adults; PEF (particles emission factor) = 1.36×10^9^m^3^ kg^−1^ for both children and adults; ESSA (exposed skin surface area) = 2800 cm^2^ for children and 5700 cm^2^ for adults; SAF (skin adherence factor) = 0.2 mg cm^−2^ for children and 0.07 mg cm^−2^ for adults; DAF (dermal absorption factor) = 0.001 for both children and adults for Cd (values for other elements are not available, therefore, 0.001 was used to normalize the CDI values); and AVT (average time for non-carcinogens) (AVT = 365 × ED) and AVT = 365 × 70 days for carcinogens.^19^,^22–24^

### Hazard quotient

Hazard quotient (HQ) and hazard index (HI) methods were used to evaluate the non-carcinogenic risk for heavy metals exposure by children and adults in the study area. The HQ is the ratio of a single metal exposure level over a specified time period to a reference dose. The hazard index is the summation of more than one HQ for multiple metals and/or exposure pathways.^18^,^25^ Hence, a combination of non-cancer risk for humans from different exposure pathways can be estimated by adding the HI of each of the exposure pathways to the deleterious metals.^26^ The total hazard quotient (THQ) and total hazard index (THI) were also considered, which are the sum of all HQ and HI for all the metals, respectively.^18^,^25^,^27^ The equations for estimating the HQ, HI, THQ and THI of the metals are based on the model proposed by the USEPA as shown in Equations 5, 6, 7 and 8, respectively.^18^

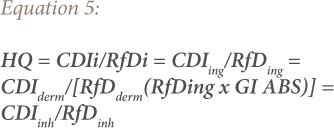


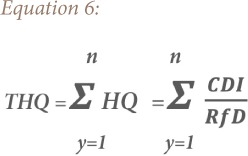


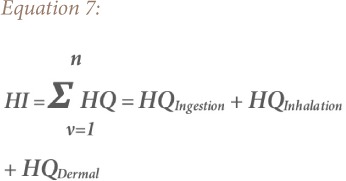


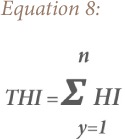
where, CDIi (mg kg^−1^ day^−1^) is the chronic daily dose of heavy metals *y*; HQ is hazard quotient of the heavy metal *y* in the soil through ingestion (CDI*_ing_*), inhalation (CDI*_inh_*) and dermal contact (CDI*_dermal_*); RfDi (mg kg^−1^ day^−1^) is reference dose of the heavy metals *y*, RfDing (ingestion reference dose), RfDderm (dermal reference dose), RfDinh (inhalation reference dose); GI ABS is the gastrointestinal absorption factor; THQ is total hazard quotient for *n* number of heavy metals; HI is hazard index of the heavy metal *y*; and THI is total hazard index for *n* number of heavy metals.^28^

## Results

Table 2 shows the percentage recovery values for the heavy metals under the experimental conditions. The recoveries of metals in the spiked sample were between 81.2 – 96.8%. Since the mean data percentage recoveries for all analytes were within an acceptable range (75–125%), the obtained data demonstrated good reliability in the present study.

**Table 2:** Percentage Recovery for Heavy Metals

**Heavy metals**	**Amount spiked (mg/kg)**	**Amount recovered (mg/kg)**	**Percentage Recovery (%)**
Cu	5.00	4.30	86.00
Fe	5.00	4.84	96.80
Cd	5.00	4.20	84.00
Pb	5.00	4.22	84.40
Zn	5.00	4.06	81.20

### Heavy metals analysis

The results of the total metal concentrations of the analyzed soils are presented in Figures 3a–b. Generally, the concentrations of metals in the transformer oil-impacted soil and control soil for both surface and subsurface soils showed that Fe had the highest mean concentration, while Cd has the lowest. In the surface soils, Fe had a mean concentration of 181.13 ± 18.71 mg/kg (transformer oil-impacted soils), and 177.5 ± 12.28 mg/kg (control soils), while Cd had a mean concentration of 1.67 ± 0.13 mg/kg (transformer oil-impacted soils), and 1.59 ± 0.20 mg/kg (control soils). In the subsurface soil, Fe had mean concentration of 177.13 ± 18.56 mg/kg (transformer oil-contaminated soils) and 179 ± 10.25 mg/kg (control soils), while Cd had a mean concentration of 1.62 ± 0.15 mg/kg (transformer oil-contaminated soils) and 1.57 ± 0.74 mg/kg (control soils).

**Figure 3a:**
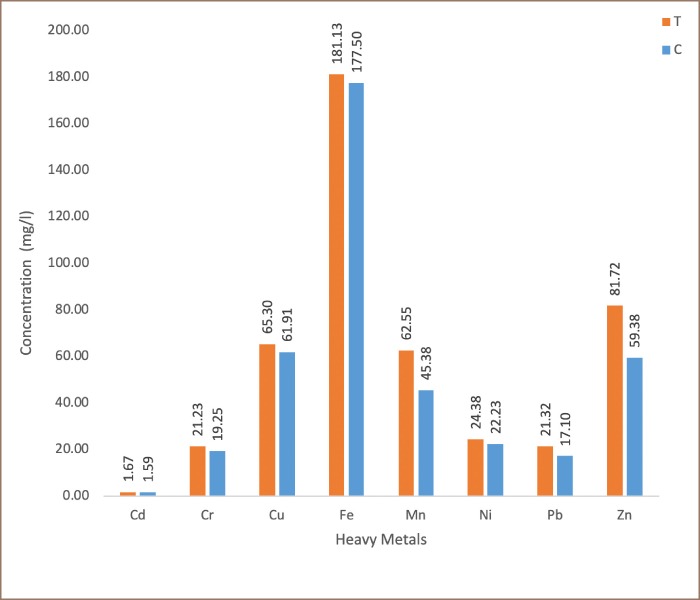
Frequency distribution of heavy metals in surface soils. Abbreviations: T, transformer oil-contaminated soil; C, control soil.

**Figure 3b:**
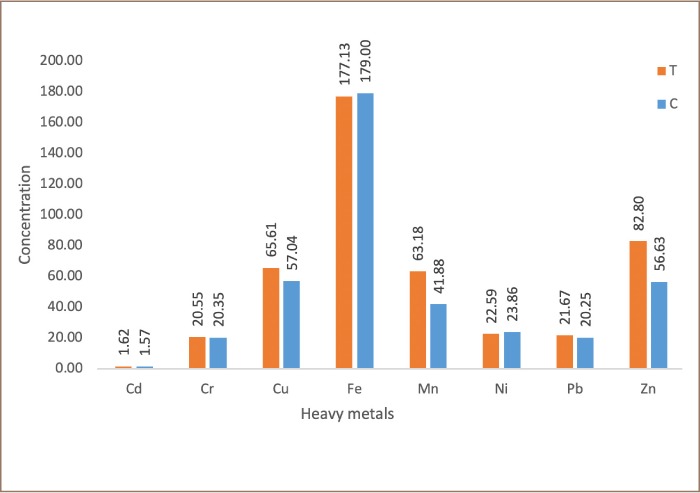
Frequency distribution of heavy metals in subsurface soils. Abbreviations: T, transformer oil-contaminated soil; C, control soil.

**Figure 4a:**
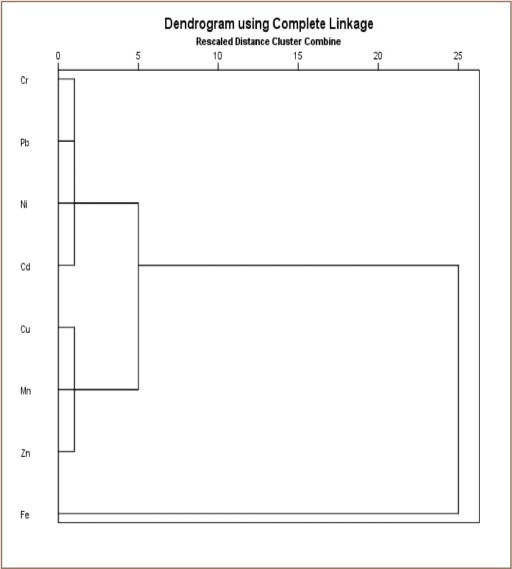
Cluster analysis results of heavy metals in surface soil of transformer oil-contaminated soil

**Figure 4b:**
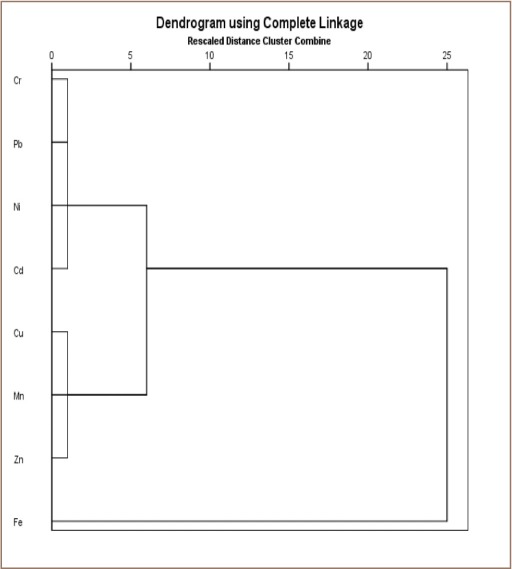
Cluster analysis of heavy metals in subsurface soil of transformer oil-contaminated soil

### Geo-accumulation index, pollution index, analysis of variance and cluster analysis of metal concentrations

The values for geo-accumulation index (I_geo_) and pollution index (PI) are listed in Table 3.

**Table 3:** Geo-accumulation Index and Pollution Index Values for Heavy Metals in Transformer Oil Soils

**Metals**	**Transformer oil-contaminated soil**	

	**Igeo**	**PI**
Cd	−0.52	0.56
Cr	−0.44	0.21
Cu	−0.51	0.65
Fe	−0.56	0.00
Mn	−0.12	0.03
Ni	−0.45	0.49
Pb	−0.27	0.21
Zn	−0.12	0.27

The results of the analysis of variance *(Table 4)* using soil depth (surface soil and subsurface soil) as the dependent variable showed the analyzed metals in the soil samples differed significantly by soil depth for Cd, Mn, Zn and total metal burden, while Cr, Cu, Fe, Ni, and Pb showed no significant difference according to soil depth.

**Table 4:** Analysis of Variance of Metals in Terms of Sample Soil Depth

**Metals**	**Depth**		**Soil samples**	
	
	**Surface soil (0 - 15 cm)**	**Subsurface soil (15 - 30 cm)**	**T**	**C**
Cd	11.05a	10.64b	16.41a	1.92b
Cr	20.51a	20.64a	20.89a	19.80a
Cu	48.68a	46.82a	65.46a	59.48b
Fe	158.65a	155.30a	179.13a	178.25a
Mn	64.73a	62.27b	62.87a	43.62b
Ni	18.60a	18.43a	23.48a	23.04a
Pb	17.00a	16.88a	21.49a	18.68b
Zn	77.05a	64.41b	82.26a	58.00b
Metal burden	416.26a	395.38b	471.98a	416.64b

Means with the same alphabet in each row are not significantly different at 5% probability according to Duncan's multiple range test.

Abbreviations: T, transformer oil-contaminated soil samples; C, control soil samples.

A summary of the comparison of potentially toxic metal concentrations in the present study with similar studies and their standard permissible limits is presented in Table 5.

**Table 5:** Comparison of Metal Concentrations (mg/kg) in Surface Soils with Similar Studies and Standard Permissible Limits

**Element (mg/kg)**	Present study^*^	**Adebiyi and Adeyemi^33^**	**Adebiyi and Afedia^34^**	**Akpan *et al.*,^35^**	**Target values**	

	**(range and mean ± SD)**				**WHO/FAO^31^**	**DPR^30^**
Fe	158.75 - 207.50 (181.13 ± 18 71)	2656–2940 (2777)	365–479 (413)	32520.0 - 85400.0 (58511.7±15635.7)	5000	-
Zn	71.10 - 92.70 (81.72 ±6.76)	836.9–1018 (910.8)	352–477 (418)	198.6 – 7019.0 (1989.1±2168.2)	300	140
Cu	51.44 - 88.45 (65.30 ± 11.64)	831.4–1050 (917.7)	138–271 (198)	180.3 - 433.8 (309.5±85.1)	100	36
Mn	54.00 - 73.80 (62.55 ± 5.87)	2368–2549 (2456)	877–1,388 (1,080)	813.5 - 6943.1 (2911.1±2355.9)	2000	-
Pb	16.97 - 24.86 (21.32 ± 2.19)	488.6–520.4 (512.1)	20.0–31.0 (26.0)	46.2 - 460.7 (98.9±163.1)	100	85
Cr	19.00 - 24.75 (21.23 ±1.93)	413.8–465.9 (451.3)	96.0–237 (169)	179.8 - 1006.3 (516.9±260.4)	100	100
Ni	20.15 - 28.28 (24.38 ±2.68)	268.7–368.7 (329.1)	101–167 (137)	126.0 - 290.2 (180.8±60.4)	50	35
Cd	1.48 - 1.88 (1.67 ±0.13)	ND	ND	ND	3	0.8

^*^ Transformer oil-contaminated soil

Abbreviations: ND, not determined; WHO, World Health Organization; FAO, Food and Agriculture Organization; DPR, Department of Petroleum Resources.

### Potential human health risk assessment

**Figure 5:**
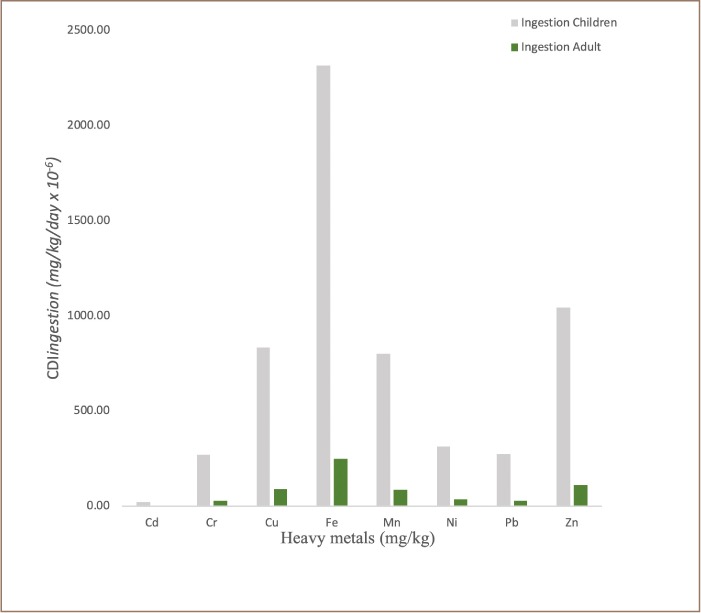
Frequency distribution of the chronic daily intake (oral) for non-carcinogenic risk for children and adults in transformer oil-impacted soils (mg/kg/day × 10^−6^)

**Figure 6:**
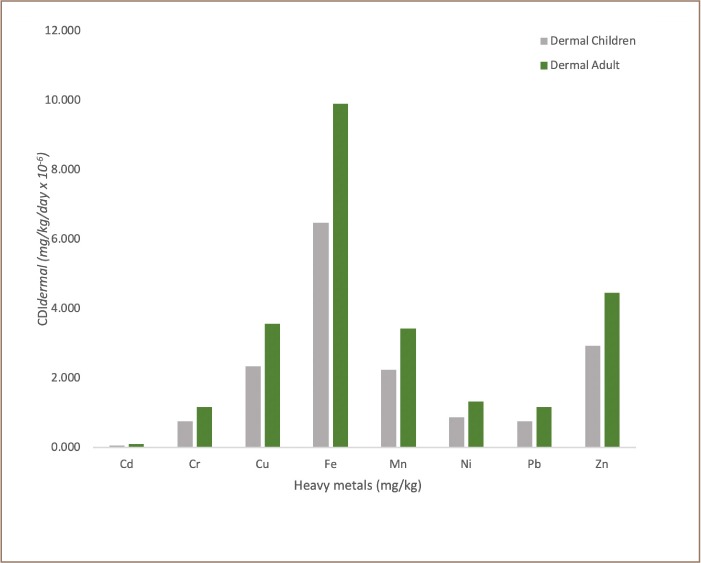
Frequency distribution of chronic daily dermal intake for non-carcinogenic risk for children and adults in transformer oil-impacted soils (mg/kg/day × 10^−6^)

**Figure 7:**
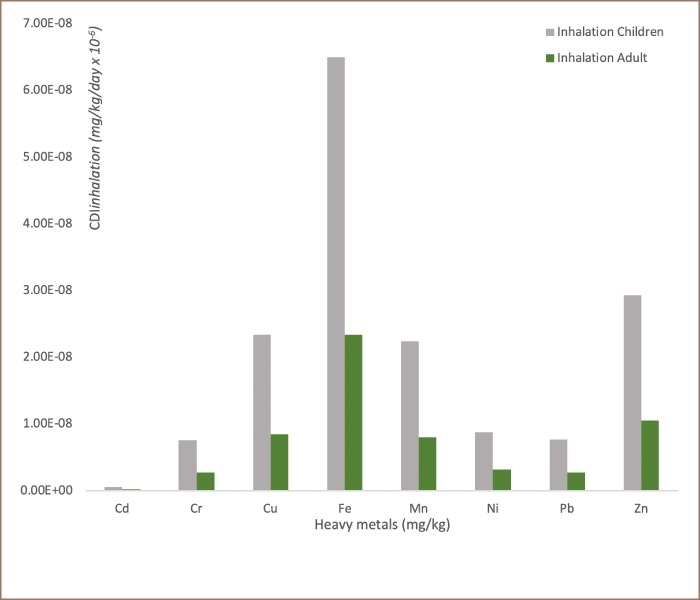
Frequency distribution of chronic daily inhalation intake for non-carcinogenic risk for children and adults in transformer oil-impacted soils (mg/kg/day × 10^−6^)

The frequency distributions of chronic daily intake (oral) for non-carcinogenic risk, chronic daily dermal intake for non-carcinogenic risk and chronic daily inhalation intake for non-carcinogenic risk for children and adults in transformer oil impacted soils are reported in Figures 5, 6 and 7, respectively. The assessment of potential human health risk consists of non-carcinogenic risk posed by heavy metals in the transformer oil-impacted soils in the study area. The chronic daily intake for non-carcinogenic risk assessment results showed the mean CDI_ingestion_, CDI_dermal_ and CDI_inhalation_ exposure of all the analyzed metals for children and adults increased in the following order: Cd < Cr < Pb < Ni < Mn < Cu < Zn < Fe. It was also observed that the chronic daily intake risk exposure pathway increased in order: CDI_inhalation_ < CDI_dermal_ < CDI_ingestion._

### Chemical partitioning and distribution of metals in soil

**Figure 8:**
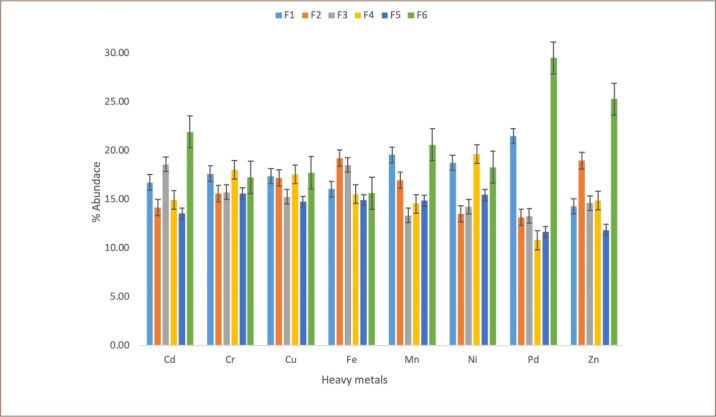
Distribution of the percentage abundance of heavy metals in the different chemical fractions of surface transformer oil-impacted soils. Abbreviations: F1, water-soluble fraction; F2, exchangeable fraction; F3, carbonate bound fraction; F4, Fe-Mn bound fraction; F5, organic bound fraction; F6, residual or inert fraction.

The results of the analysis of chemical speciation of metals in the study soils based on their geochemical fractions are summarized in Figure 8.

#### Cadmium

The results of the speciation analysis of Cd showed that the mean levels of the metal in the various fractions of the surface soil of the transformer oil impacted soil decreased in the order of: residual > carbonate-bound > water-soluble > Fe-Mn oxide-bound > exchangeable > organic matter-bound; while the mean levels of Cd in the various fractions of subsurface soil decreased in the order of: residual > carbonate-bound > water-soluble > organic matter-bound > exchangeable > Fe-Mn oxide-bound.

#### Chromium

The results of the speciation analysis of Cr showed that the mean levels of the metal in the various fractions of the surface soil of the transformer oil impacted soil decreased in the order of: Fe-Mn oxide-bound > water-soluble > residual > carbonate-bound > exchangeable > organic matter-bound; while the mean levels of Cr in the various fractions of subsurface soil decreased in the order of: carbonate-bound > exchangeable > water-soluble > Fe-Mn oxide-bound > organic matter-bound > residual.

#### Copper

The results of the speciation analysis of Cu showed that the mean levels of the metal in the various fractions of the surface soil of the transformer oil impacted soil decreased in the order of: residual > Fe-Mn oxide-bound > water-soluble > exchangeable > carbonate-bound > organic matter-bound; while the mean levels of Cu in the various fractions of subsurface soil decreased in the order of: water-soluble > exchangeable > organic matter-bound > Fe-Mn oxide-bound > carbonate-bound > residual. The residual fraction had the highest amount of Cu.

#### Iron

The result of the speciation analysis of Fe showed that the mean levels of the metal in the various fractions of the surface soil of the transformer oil impacted soil decreased in the order of: exchangeable > carbonate-bound > water-soluble > residual > Fe-Mn oxide-bound > organic matter-bound; while the mean levels of Fe in the various fractions of subsurface soil decreased in the order of: Fe-Mn oxide-bound > carbonate-bound > water-soluble > exchangeable > residual > organic matter-bound.

#### Manganese

The results of the speciation analysis of Mn shows that the mean levels of Mn in the various fractions of the surface soil of the transformer oil impacted soil decreased in the order of: residual > water-soluble > exchangeable > Fe-Mn oxide-bound > organic matter-bound > carbonate-bound; while the mean levels of Mn in the various fractions of subsurface soil decreased in the order of: residual > organic matter-bound > water-soluble > exchangeable > carbonate-bound > Fe-Mn oxide-bound.

#### Nickel

The results of the speciation analysis of Ni shows that the mean levels of the metal in the various fractions of the surface soil of the transformer oil impacted soil decreased in the order of: Fe-Mn oxide-bound > water-soluble > residual > organic matter-bound > carbonate-bound > exchangeable; while the mean levels of Ni in the various fractions of subsurface soil decreased in the order of: residual > organic matter-bound > water-soluble > exchangeable > carbonate-bound > Fe-Mn oxide-bound. The concentration of nickel in the transformer oil impacted soil was highest in the Fe-Mn oxide fraction and water-soluble fraction.

#### Lead

The results of the speciation analysis of Pb showed that the mean levels of the metal in the various fractions of the surface soil of the transformer oil impacted soil decreased in the order of: residual > water-soluble > carbonate-bound > exchangeable > organic matter-bound > Fe-Mn oxide-bound; while the mean levels of Pb in the various fractions of subsurface soil decreased in the order of: residual > water-soluble > Fe-Mn oxide-bound > carbonate-bound > exchangeable > organic matter-bound. There was more Pb in the residual fractions than in the other fraction, and there was also a significant percentage present in the carbonate-bound and water-soluble fractions for the surface soils. In the subsurface soils there was a significant percentage of Pb in the water-soluble and Fe-Mn oxide fractions.

#### Zinc

The results of the speciation analysis of Zn showed that the mean levels of the metal in the various fractions of the surface soil of the transformer oil-impacted soil decreased in the order of: residual > exchangeable > Fe-Mn oxide-bound > carbonate-bound > water-soluble > organic matter-bound; while the mean levels of Zn in the various fractions of subsurface soil decreased in the order of: residual > carbonate-bound > water-soluble > Fe-Mn oxide-bound > organic matter-bound > exchangeable.

### Mobility factor of heavy metals across soil samples

The mobile fractions (water-soluble (F1), exchangeable (F2) and carbonate (F3)) of the metals in the oil-impacted soils are metals that can lead to human exposure through ingestion and are usually considered to have anthropogenic origins.^29^ These fractions, which were determined on the basis of absolute and relative index values, are weakly bound to soil components, and are the most reactive, mobile and potentially available/bioavailable fractions.^29^ The results of the analysis of the mobility factor of the analyzed metals in the transformer oil soils are presented in Table 6. The mobility factors of the metals in the surface soil of the transformer oil-impacted soils were observed to decrease in the following order: Fe > Mn > Cd > Cu > Cr > Pb > Zn> Ni, while the mobility factor of the sub-surface soil of the transformer oil-impacted soil samples decreased in the following order: Cu > Cr > Fe > Cd > Ni > Zn > Pb > Mn. Mobility factors of the analyzed metals in the transformer oil-impacted soils were observed to decrease with an increase in depth, except for Cr, Cu and Ni.

**Table 6:** Mobility Factor of Metals in Transformer Oil-Impacted Soil by Sample Depth (cm)

**Sample location code**	**0–15cm**	**15–30 cm**	**0–15cm**	**15–30 cm**	**0–15cm**	**15–30 cm**	**0–15cm**	**15–30 cm**	**0–15cm**	**15–30 cm**	**0–15cm**	**15–30 cm**	**0–15cm**	**15–30 cm**	**0–15cm**	**15–30 cm**
**TO1**	55.35	46.39	48.33	67.83	60.38	57.89	52.91	46.66	60.55	34.01	38.92	32.32	51.12	23.91	40.96	33.75
**TO2**	50.63	44.30	58.63	65.37	45.36	45.01	61.48	58.48	37.22	48.15	52.60	30.84	55.03	59.12	48.91	40.21
**TO3**	57.54	45.11	44.75	38.70	48.75	43.08	60.90	58.57	44.96	33.53	43.79	60.26	43.34	40.22	56.60	51.69
**TO4**	46.62	50.63	47.54	58.63	54.84	56.00	48.20	41.02	44.09	35.65	49.48	57.41	42.53	36.69	41.76	56.41
**TO5**	24.54	60.12	42.77	51.03	46.68	59.66	47.31	59.35	60.28	43.41	38.60	55.67	57.10	62.31	47.13	54.74
**TO6**	42.55	45.02	45.35	62.57	50.00	59.26	55.81	46.94	49.07	47.06	52.67	51.75	59.42	47.01	52.87	54.22
**TO7**	57.18	34.88	48.09	50.41	53.42	61.11	48.23	41.17	62.13	41.13	43.82	36.91	30.81	50.46	37.18	39.80
**TO8**	52.08	54.05	38.78	46.86	45.16	57.14	62.33	52.12	56.35	45.11	33.23	35.24	36.69	39.72	55.66	46.60
**TO9**	67.03	46.51	62.56	59.07	42.59	52.70	47.64	55.87	49.84	33.11	59.22	45.95	54.67	37.19	48.24	47.67
**TO10**	44.64	49.02	54.87	34.06	50.88	44.44	52.02	54.02	34.68	56.08	55.13	62.55	49.61	25.32	45.35	40.91
**Mean**	49.82	47.60	49.17	53.45	49.81	53.63	53.68	51.42	49.92	41.72	46.75	46.89	48.03	42.20	47.47	46.60
**SD**	11.43	6.65	7.37	11.27	5.33	6.92	6.08	7.05	9.79	7.66	8.37	12.21	9.40	12.77	6.39	7.69

Abbreviations: TO, transformer oil-contaminated soil sampling locations; SD, standard deviation.

## Discussion

Interpretation of the I_geo_ results was performed based on the scale proposed and adapted by Asubiojo *et al*.^13^ The I_geo_ values were generally low for all the metals with I_geo_ < 0 (unpolluted) in all the samples, while the PI values were also less than 1 (PI < 1) for all the metals in the transformer oil-impacted soils. These low PI values demonstrate that the contributions of anthropogenic activities to the number of metals in the contamination site may be insignificant.

The significant differences between soil samples according to soil depth in the results of the analysis of variance are likely due to differences in the mobility and bioavailability of the metals in the soils and differences in the background levels of the metals in different sample locations. Similarly, using soil sample type (i.e. transformer oil-impacted soils and control soils) as the dependent variables, the concentrations of analyzed metals in the soils differed significantly across soil types for Cd, Cu, Mn, Pb, Zn, and total metal burden. For Fe and Ni, there was no significant difference between the transformer oil-contaminated soils and control soils. This indicates non-uniformity in the levels of the metals found in the different sampling locations. This could be due to uneven anthropogenic inputs of the metals, differences in the level of contribution of metals from the oil to the soil, and the lithogenic composition of the different locations, suggesting that the metals might not originate from the same source.

Cluster analysis of the metal concentrations showed a fair correlation among some metals. Cadmium, Cr, Cu, Fe, Mn, Ni, and Zn are transition metals with similar properties, such as variable oxidation state among other properties, and are known to be associated with petroleum hydrocarbon formation.^5^ Lead is used in the manufacturing of high voltage generators and transformers. Iron, Cu, Zn, and Mn are essential metals vital for growth and plant life and are relatively abundant in the earth's crust, hence their close clustering.

### Comparison of heavy metal concentrations with similar studies and standard permissible limits

The metal concentrations in the present study fell below the standard permissible limits set by the Department of Petroleum Resources, and World Health Organization/Food and Agriculture Organization, with the exception of Cd and Cu, which had a mean value higher than the permissible limit set by the Department of Petroleum Resources. 30,31 High concentrations of Cd could pose a health risk, as it tends to accumulate in the human body.^32^ The total metal concentrations obtained in this study are comparatively lower than those reported by Adebiyi and Adeyemi, who reported concentrations of heavy metals in the soils around a petroleum product depot; similar to a study by Adebiyi and Afedia, who reported concentrations of heavy metals and the impact of used petrochemical oils on soil properties with special reference to the physicochemical properties of soils around automobile repair workshops; and Akpan *et al*., who reported concentrations of heavy metals in municipal surface soils polluted with spent crude oil products.^33–35^ This variation is probably due to the differences in the source and/or degrees of contamination.

### Potential hazard assessment

Human exposure (adults and children) to heavy metals and other environmental pollutants in soils can occur through the following three main pathways: direct ingestion of soil dust particles (CDI_ingestion_), inhalation of soil suspended particles through mouth and nose (CDI_inhalation_), and dermal absorption of heavy metals in soil particles adhered to exposed skin (CDI_dermal_). In this study, the lifetime exposure risk assessment to heavy metals in soils contaminated with transformer oil through these pathways was carried out using accepted model proposed by the USEPA.^18^

Comparison of exposure pathways indicated that soil ingestion was the most significant contributor to total health risk. The HQs associated with the ingestion of heavy metals for children were significantly greater than those for adults. The risk from soil ingestion was 10 times greater than that of inhalation and dermal exposure; thus, this factor should be considered during health risk assessments. In all chronic daily intake risk exposure pathways (CDI_ingestion_, CDI_inhalation_ and CDI_dermal_), the values obtained for the analyzed metals were higher for children than adults. This suggests that children in environments contaminated with transformer oil are likely to be in danger of being exposed to high doses of toxic metals if the contaminated soil/dust is orally ingested or inhaled or comes into contact with their skin.

Hazard quotient and HI methods were applied to evaluate the human health risk of heavy metal exposure from the transformer oil-impacted soils in the present study *(Tables 7 and 8).*

**Table 7:** Hazard Quotient for Children in Transformer Oil-Impacted Soil Samples

**Metals**	**Cmetal**	**CDI_ingestion_ (mg/kg day)**	**CDI_dermal_ (mg/kg day)**	**CDI_inhalation_ (mg/m^3^)**	**RfD_ingestion_ (mg/kg day)**	**RfD_dermal_ (mg/kg day)**	**RfD_inhalation_ (mg/m^3^)**	**HQ_ingestion_**	**HQ_dermal_**	**HQ_inhalation_**	**HI**
Cd	1.67	2.13E-04	5.96E-07	7.83E-10	1.00E-01 ^a^	2.50E-05 ^a^	1.00E-03 ^b^	2.13E-03	2.38E-02	7.83E-07	2.6OE-02
Cr	21.23	2.71E-03	7.60E-06	9.98E-09	3.00E-03 ^a^	7.50E-05 ^a^	2.86E-05 ^b^	9.05E-01	1.01E-01	3.49E-04	1.01E+00
Cu	65.30	8.35E-03	2.34E-05	3.07E-08	4.00E-02 ^a^	4.00E-02 ^a^	4.02E-02 ^b^	2.09E-01	5.84E-04	7.64E-07	2.09E-01
Fe	181.13	2.32E-02	6.48E-05	8.51E-08	7.00E-01 ^a^	7.00E-01 ^a^	7.00E-05 ^c^	3.31E-02	9.26E-05	1.22E-03	3.44E-02
Mn	62.55	8.00E-03	2.24E-05	2.94E-08	1.40E-01 ^a^	1.40E-01 ^a^	5.00E-02 ^b^	5.71E-02	1.60E-04	5.88E-07	5.73E-02
Ni	24.38	3.12E-03	8.73E-06	1.15E-08	1.10E-02 ^a^	4.40E-04 ^a^	2.06E-02 ^b^	2.83E-01	1.98E-02	5.56E-07	3.03E-01
Pb	21.32	2.73E-03	7.63E-06	1.00E-08	3.00E-01 ^a^	3.50E-02 ^a^	3.52E-03 ^b^	9.09E-03	2.18E-04	2.85E-06	9.31E-03
Zn	81.72	1.04E-02	2.93E-05	3.84E-08	3.00E-01 ^a^	3.00E-01 ^a^	3.00E-01^b^	3.48E-02	9.75E-05	1.28E-07	3.49E-02
THQ								1.53E+00	1.46E-01	1.57E-03	-
THI											1.68E+00

^a^ USEPA (2015), Olujimi *et al.* (2015).^20^,^28^

^b^ USEPA (2018), Adedeji *et al.* (2019).^44^,^45^

^c^ United States Department of Energy (2011), Gbadamosi (2018).^24^,^46^

Abbreviations: Cmetal, concentration of analyzed metals; Rfd, reference dose

**Table 8:** Hazard Quotient for Adults in Transformer Oil-Impacted Soil Samples

**Metals**	**Cmetal**	**CDI_ingestion_ (mg/kg day)**	**CDI_dermal_ (mg/kg day)**	**CDI_inhalation_ (mg/m^3^)**	**RfD_ingestion_ (mg/kg day)**	**RfD_dermal_ (mg/kg day)**	**RfD_inhalation_ (mg/m^3^)**	**HQ_ingestion_**	**HQ_dermal_**	**HQ_inhalation_**	**HI**
Cd	1.67	2.28E-05	9.10E-08	3.35E-10	1.00E-01 ^a^	2.50E-05 ^a^	1.00E-03 ^b^	2.28E-04	3.64E-03	3.35E-07	3.87E-03
Cr	21.23	2.91E-04	1.16E-06	4.28E-09	3.00E-03 ^a^	7.50E-05 ^a^	2.86E-05 ^b^	9.69E-02	1.55E-02	1.50E-04	1.13E-01
Cu	65.30	8.95E-04	3.57E-06	1.32E-08	4.00E-02 ^a^	4.00E-02 ^a^	4.02E-02 ^b^	2.24E-02	8.92E-05	3.27E-07	2.25E-02
Fe	181.13	2.48E-03	9.90E-06	3.65E-08	7.00E-01 ^a^	7.00E-01 ^a^	7.00E-05 ^c^	3.54E-03	1.41E-05	5.21E-04	4.08E-03
Mn	62.55	8.57E-04	3.42E-06	1.26E-08	1.40E-01 ^a^	1.40E-01 ^a^	5.00E-02 ^b^	6.12E-03	2.44E-05	2.52E-07	6.15E-03
Ni	24.38	3.34E-04	1.33E-06	4.91E-09	1.10E-02 ^a^	4.40E-04 ^a^	2.06E-02 ^b^	3.04E-02	3.03E-03	2.38E-07	3.34E-02
Pb	21.32	2.92E-04	1.17E-06	4.29E-09	3.00E-01 ^a^	3.50E-02 ^a^	3.52E-03 ^b^	9.73E-04	3.33E-05	1.22E-06	1.01E-03
Zn	81.72	1.12E-03	4.47E-06	1.65E-08	3.00E-01 ^a^	3.00E-01 ^a^	3.00E-01 ^b^	3.73E-03	1.49E-05	5.49E-08	3.75E-03
THQ								1.64E-01	2.23E-02	6.73E-04	-
THI											1.87E-01

^a^ USEPA (2015), Olujimi *et al.* (2015).^20^,^28^

^b^ USEPA (2018), Adedeji *et al.* (2019).^44^,^45^

^c^ United States Department of Energy (2011), Gbadamosi (2018).^24^,^46^

Abbreviations: Cmetal, concentration of analyzed metals; Rfd, reference dose

The results of the present study indicate that the HQ for all metals was less than 1. The HI for all metals in transformer oil-impacted soil was less than 1, except for Cr for children, which showed HI > 1. For metals which showed HQ and HI values less than 1, there was no risk posed to the health of children or adults by the metals. Exposure to metals with high THI values in contaminated soil is of great concern for both adults and children. According to the USEPA, adverse health effects are unlikely to occur if HQ < 1.^18^ However, if HQ > 1, adverse health effects are likely and HQ > 10 indicates high chronic risk, while THI values > 1 implies a significant probability of non-carcinogenic effects and THI < 1 indicates low probability of noncarcinogenic effects. Additionally, THI between 1–10 indicates moderate hazard, while THI > 10 indicates high hazard.^36^ According to Agomuo and Amadi, the higher the THI value, the higher the probability of experiencing long term health problems with associated toxicities.^37^ However, to prevent the bioaccumulation of these metals in both plants and organisms, it is necessary to determine their availability and health-related problems.

Comparing THI values for children and adults revealed that children have higher chances of non-carcinogenic and/or harmful health risks arising from exposure to heavy metals in the oil impacted soils than adults, although the calculated heavy metals exposure risks associated with contaminated soils showed a high degree of uncertainty.^25^ As such, the risk evaluation based on the total concentrations of heavy metals in the contaminated soils may overestimate the actual hazards they pose to humans. Considering the hazard index of a contaminated environment, Grzetic and Ghariani noted health hazards arising from exposure to heavy metals are common as a result of the cumulative effects of different heavy metals.^21^ This is a major concern to environmental toxicologists as it affects the health of both children and adults.

### Chemical partitioning and distribution of metals in soil

Cadmium was found to be more greatly associated with the residual fraction. This indicates that Cd does not generally constitute an environmental risk as a result of the stable nature of the compound and the fact that the metals are bonded firmly within a mineral lattice that restricts its bioavailability. In addition, a significant portion was in the organic matter-bound and Fe-Mn oxide fractions of the surface and subsurface soils, respectively. Metals have an affinity for organic complexes and Fe-Mn oxide precipitates this, due to the presence of active sites on which the metals absorb, thereby reducing their mobility and bioavailability. These metals could be made mobile by activities of microorganisms, which have reducing or oxidizing potentials, releasing the metals into the soluble fraction.^38^ Cadmium associated with the non-residual fraction, especially the water-soluble fraction, may be easily taken up by plants growing in the soil, thereby posing a human health risk.^39^

#### Chromium

The high presence of Cr in water-soluble and organic matter bound fractions in the oil-impacted soils and the high concentration of the metal in the Fe-Mn oxide fraction and water-soluble fraction provides information about the relative stability of the metal in the petroleum hydrocarbon-impacted soil. The concentration of Cr in the water-soluble fraction was high, hence the metal tends to be mobile and available for uptake. These results are in agreement with the study of Peter *et al.* on the chemical fractionation of heavy metals in the soil of auto-mechanic workshops in Akure, Nigeria, which reported low concentrations of Cr in exchangeable and carbonate fractions of soil, whereas in the control soil samples the concentration of Cr in the exchangeable and carbonate fraction was high.^40^ The source of Cr in both soils is mainly natural, with little anthropogenic input. The concentrations of Cr found in the residual fraction are, however, not likely to enter the food chain since the residual fraction is very stable, less reactive and less bioavailable. Heavy metals found in this matrix are believed to be trapped and occluded within the crystal lattice of a layer of silicate and well-crystallized oxide minerals.^9^,^17^

#### Copper

The relationship between Cu and the Fe-Mn oxide fraction in this study is similar to that of Peter *et al*. who noted that this relationship could be a result of the tendency of Cu complexes in the organic fraction to have high formation constant.^39^ The low presence of Cu in the carbonate and organic matter bound fraction could be a result of high stability of Cu in the crystal matrix of the soil and in the water-soluble fraction. However, Cu tends to form a complex with organic matter and therefore can be leached into the environment under oxidizing conditions. This complex is generally described by an ion-exchange/organometallic complexation. Carbonyl, phenolic and carboxylic functional groups of humic and fulvic acid are assumed to be responsible for this binding. The Cu in the Fe-Mn oxide fraction is not considered very available, as it is thought to be associated with stable, high molecular weight, and humic substances that slowly release small amounts of metal.^28^ The occurrence of Cu in the soil could be attributed to both natural and anthropogenic sources.

#### Iron

The majority of Fe in the oil-impacted soils was associated with exchangeable, carbonate and water-soluble fractions, which is commonly considered a mobile phase. Hence, it is available for uptake by plants in this soil. The substantial amount of iron in the Fe-Mn oxide fraction could be attributed to the transformation of Fe^2+^ into Fe^3+^ which is easily precipitated as Fe oxyhydroxide under strongly oxidizing conditions and neutral pH values.^40^ The relatively low amount of Fe in the water-soluble fraction could be a result of the high tendency for Fe (an essential element) to be easily absorbed and utilized by plants and other organisms in the soil environment.^40^ The concentrations of Fe in the residual and Fe-Mn oxide fraction in soils were high, hence the metal could be considered relatively stable, slowly mobile and poorly available, but could change with variations in redox conditions. This result is in agreement with the study of Peter *et al*. who reported low concentrations of iron in exchangeable and carbonate fractions of soil and sediment samples.^39^ The obtained Fe results also revealed that it could originate from anthropogenic sources at the study area.

#### Manganese

The presence of Mn in the organic bound fraction could probably be as a result of its tendency to form complexes between the metal and natural organic matters like humic and fulvic acids. It was observed in the study that the content of Mn in the residual and Fe-Mn oxide fractions was low. According to Ogunfowokan *et al.* the structure of a mineral's crystal in soil has poor association or retention ability, hence the low content of Mn in these fractions.^15^

#### Nickel

A significant fraction of Ni was present in the organic matter-bound and residual fractions, suggesting that this metal might have resulted from a natural source.^38^ Generally, the Ni concentration was low in the carbonate-bound fraction and this is consistent with studies by Osakwe and Okolie.^40^ The low amount of Ni in the exchangeable and carbonate fraction could also be attributed to the high presence of organic matter resulting from the petroleum hydrocarbon product.

#### Lead

The levels of Pb in the present study are consistent with a report by Osakwe.^17^ The substantial amount of Pb in the Fe-Mn oxide phase has been attributed to the sorption of Pb to Mn and Fe oxides.

#### Zinc

The presence of a substantial amount of Zn in the mobile fractions of the control soil samples provides information about its availability compared to the transformer oil-impacted soils. Zinc is highly retained in the residual fraction in all soils, suggesting that it is strongly bonded to the crystal lattices of minerals. The high concentrations of metals in the residual fraction are assumed to be less available compared to those in the non-residual fraction, and therefore present less toxicity in terms of environmental pollution.^7^

### Heavy metals mobility factors across soil samples

The high mobility factors of Fe and Mn in the surface samples of the transformer oil impacted soil reveals that they are of anthropogenic origin with high potential to be mobile and bioavailable for uptake by plants and living organisms.^15^ A high mobility factor value for a heavy metal shows relative high lability and bioavailability of the metal in the soil.^42^ In addition, it provides information about the levels of exchangeable and carbonate-bound metals, as well as the vulnerability of living things to heavy metals in the study area. Chronic exposure can arise from a single contamination event, and this exposure could be hazardous to soil microbial activities and soil productivity.^42^

Generally, the mean values of the mobility factors for all metals in transformer oil impacted soils were less than 60%. This is an indication of moderate stability of the metals in the soil samples and might be a result of the presence of organic matter content in the soil which forms insoluble complex compounds and tends to limit the mobility of heavy metals.^39^ According to Salbu and Oughton, heavy metals are potentially available for plant uptake if their mobility factor is above 10%.^43^ The mobility factors of the heavy metals in the study area were above 10%, hence they have high availability. The bioavailability and transportation of heavy metals in the soil are influenced by several factors such as soil pH, organic matter content, amounts and forms of oxides and carbonates, effective cation exchange capacity, and mineral composition.^39^

## Conclusions

The impact of transformer oil on the soils in the study areas was determined to assess the environmental and health implications of oil-impacted soils. Various statistical approaches were employed in order to assess the distribution and chemical forms of these soils. The PI and I_geo_ revealed values less than unity for all the analyzed metals, indicating that the analyzed soils were not polluted with the heavy metals, but were contaminated, reflecting anthropogenic influences. The mean values of Cd were higher than the permissible limit set by the Department of Petroleum Resources and this is an environmental concern, considering the health risk associated with elevated concentrations of metals in the environment.^30^ The chemical fractionation provides information on the mobility and bioavailability of these heavy metals. The clustering analysis results showed the correlation between the metals indicating chemical affinity and/or common sources. Although the analyzed metal concentrations were below their permissible limits, their chronic daily dosage was significant and may cause health hazards to humans with long term exposure. In particular, children in contaminated environments are more vulnerable to higher doses of these toxic metals via all of the chronic daily intake exposure pathways compared to adults.

Based on the findings of this study, it is recommended that high voltage transformer installation sites be designed with a polyethylene-lined pit beneath the transformer where leakages of oil can be trapped before entering the soil. Leaks of petroleum products should be reported immediately. Soil cleanup efforts are urgently needed to prevent contamination of surrounding ecosystems.
